# Are We Better-Off? The Benefits and Costs of Australian COVID-19 Lockdown

**DOI:** 10.3389/fpubh.2021.798478

**Published:** 2021-12-02

**Authors:** Anton Pak, Oyelola A. Adegboye, Emma S. McBryde

**Affiliations:** ^1^Centre for the Business and Economics of Health, The University of Queensland, Brisbane, QLD, Australia; ^2^Australian Institute of Tropical Health and Medicine, James Cook University, Townsville, QLD, Australia; ^3^Public Health and Tropical Medicine, College of Public Health, Medical and Veterinary Sciences, James Cook University, Townsville, QLD, Australia

**Keywords:** SARS-CoV-2, COVID-19, lockdown, cost-benefit, Australia

## Introduction

When compared with other countries, Australia has fared much better in COVID-19 outcomes, having experienced low COVID-19 cases, hospitalisations, and deaths. Although it is difficult to know with certainty what and to what degree led to these advantageous outcomes, many attributed this success to the early implementation of strict border closure limiting cross-border transmission and being an Island nation (1–3). Australia has been proceeding with the elimination strategy aiming to contain and crush emerging outbreaks quickly through a suite of public health interventions, with lockdowns playing a central role. However, as vaccination rates continue to rise in Australia, we opine that the lockdowns and other stringent non-pharmaceutical interventions should be phasedown as the cost to the individuals, community, and the economy is likely to outweigh the benefits of these restrictions.

At the beginning of the pandemic, most countries followed and defended the implementation of lockdowns, with the early calculations suggesting that benefits far outweigh the costs (3–5). Some empirical studies also observed heterogeneity in the effectiveness of lockdowns and advocated for a careful consideration of demographic, economic, and societal factors before implementing stay-at-home orders, especially in developing countries in which many people rely on day-to-day economic resources (6, 7). However, using more recent data, others provided a different assessment arguing that lockdowns cause more harm than good even in developing countries—with the benefit-cost ratio being significantly overestimated (8, 9). Considering the burden of prolonged lockdown that Sydney and Melbourne have been experiencing and taking into account the increasing vaccination rates across the country, our governments need to carefully consider when and how to lift lockdown and other restrictions, as there is no doubt the cost of getting this wrong is very high.

Following a critical review by Allen (10), we discuss the issues associated with the evaluation of lockdown costs and benefits and provide an opinion on lockdowns doing potentially more harm than good as Australia achieves high vaccination rates. This may be useful in timely discussions among the public, media, public health officials, and decision-makers.

### Issues in Cost-Benefit Analyses

Firstly, by following an intuitive argument that lockdowns reduce transmission of the COVID-19 infection, the direct benefits should include the reduction in the number of cases, hospitalisations, and deaths. However, by how much? To answer this, the counterfactual scenarios that should be explored are those that would represent what would have been the level of disease burden if lockdown hadn't been implemented. One must be cautious with the studies that used the “Do-nothing” or unrealistic counterfactuals, which significantly inflate the benefits of lockdowns. As we have seen, almost all, if not all, countries have implemented various other control and suppression measures, and individuals changed their behavior voluntarily in order to reduce the transmission of COVID-19 infection (11).

Secondly, several studies evaluated the impact of lockdown through changes in COVID-19 basic or effective reproduction number, which describe the contagiousness or transmissibility of COVID-19 infectious agents in epidemiological models (12, 13). These models do not take into account the fact that individuals change their behavior endogenously and can respond to the rising risks of being infected. Ignoring these endogenous individual adjustments is likely to overestimate the lockdown benefits in terms of the number of daily cases, hospitalisations, and deaths.

Thirdly, relying on the number of deaths averted due to the lockdown as a key input in the benefits calculations is not enough. In the case of COVID-19, clinical evidence indicates that elderly (and nursing home residents) and those with co-morbidities are at significantly higher risk of experiencing adverse COVID-19 health outcomes, including death (14, 15). Consequently, while reporting the number of deaths is an important indicator, the cost-benefit analysis would benefit from focusing on the residual life-expectancy of the average COVID-19 death, which then can be used to infer the number of life-years saved due to the lockdown.

Lastly, to estimate the benefits of lockdown in monetary terms, many studies relied on the economic concept of the value of a statistical life (VSL), which is calculated by observing individual willingness-to-pay for a (small) reduction in mortality risk (5, 16, 17). Population-average VSL estimates generally are elicited by examining the trade-off between wages and occupational hazards among working adults—excluding those not in the labor force. Using these population-average VSL estimates (seen in many earlier works) is hardly appropriate and may significantly overestimate the benefits since the elderly and those with co-morbidities experience higher fatality risks and generally have lower VSL. Another difficulty in identifying appropriate VSL estimates to COVID-19 deaths is that the VSL for the elderly group is highly uncertain. This makes it critical to explore the sensitivity of the results to VSL estimates and associated COVID-19 attributes that may influence them.

Apart from the issues raised in evaluating the benefits, the calculation of costs has also been problematic and often does not correspond to the full burden of prolonged lockdowns a society experience. Most studies evaluated the costs of lockdown through the loss of GDP, or in other words, the lost output, and foregone services. From a practical perspective, this is a straightforward approach due to the data availability, including high-frequency datasets from private companies. Similar to the evaluation of the benefits, the selection of the counterfactual is important to calculate the costs, as some of the costs would have occurred due to other restrictions and voluntary behavior changes.

In addition to GDP losses, lockdowns are associated with significant societal, health, and economic costs—which needs to be taken into account, especially if the VSL approach is used. If the evaluation of lockdown benefits relies on a dollar measure of utility people derive from living, then the costs should also be guided by the losses in utility; hence focusing only on the GDP losses would largely underestimate the “true” costs of lockdowns. Some of the non-monetary but tangible impacts include losses in human capital due to schools' closure and educational disruptions, losses in health outcomes due to delayed medical procedures, and losses in mental and physical well-being due to increased anxiety levels, domestic violence, and lack of physical exercise. It is challenging to be comprehensive and account for all costs due to the lockdown, but by evaluating more significant and visible of those costs, one can get closer to the actual cost of lockdown or at least establish a lower bound for the costs and more carefully compare it with the benefits.

Worth also considering in decision-making is the equity concerns with respect to who benefits and who suffers in the prolonged lockdowns. Epidemiological evidence suggests that young people are much less likely to have health consequences from COVID-19, but experience higher costs of lockdowns in terms of lost educational and employment opportunities, lost social connections, and an increased risk of adverse effects on their mental health (18–20).

## Discussion

Evaluating the costs and benefits of lockdowns in the Australian context is particularly important. It is one of the very few countries which has pursued the elimination strategy (successfully for the most part) and now trying to shift from stringent public health measures as the vaccination rates rise. Complementing timely public health response to the pandemic, Australians have shown high compliance and trust in government and health services (21). Together with the government, they have been adjusting quickly to changing environment (especially during the outbreaks). Accounting for these endogenous changes in behavior regarding increasing or decreasing risks of getting infected helps avoid the overestimation of the lockdown benefits.

Lockdown benefits significantly depend on whose lives we are saving and look at the residual life-expectancy of the average COVID-19 death. Lally (22) estimated this measure lies between 4.7 and 5.5 years for Australia using the data from the first waves of the pandemic when most of the deaths occurred among nursing home residents and those with multiple co-morbidities. In the current context of the delta strain affecting the population more generally, especially once the lockdown restrictions are lifted, these estimates will require upward adjustment. Conservative estimates would be around 10–12 years in residual life expectancy using data from the U.S. and Sweden, where the virus had been established in the community (22, 23). Furthermore, it may also be reasonable to adjust these estimates downward to account for the quality of those residual years.

With respect to the costs, capturing non-economic costs of the pandemic is an important and challenging task. Costs associated with depression, anxiety, and well-being have represented a significant burden of the lockdown. In Australia, recent research supports these findings that there was a considerable decline in community mental health in adults due to the pandemic-related restrictions (24).

## Conclusion and Recommendations

In light of the futility and detrimental impacts of lockdowns and increasing vaccination rates across the country, we recommend:

- A shift from a zero-covid policy to a COVID-19 management plan which should outline the details of how and at what stages the lockdown and other public health restrictions would be lifted.
- Moving forward, the use of lockdowns or movement restrictions should be limited and target only outbreaks in areas with a high proportion of unvaccinated people, particularly in disadvantaged and remote communities.
- Shift away from reporting COVID-19 cases and focus on reporting geographically specific health system capacity and deaths once the lockdown approach is phased out. This should be a part of the COVID-19 management plan preparing the community to live with COVID-19.
- When evaluating the impact of lockdowns retrospectively and for future planning, a broad societal perspective should be adopted to estimate the benefits and costs of lockdown.
- Also, a systematic approach to evaluate lockdowns is needed, which will allow for updating the benefit-cost ratio more frequently in response to the changes in epidemiological and economic situations.

There is no illusion that there are trade-offs, but the question that begs an answer is whether we can be better off as a community without lockdown restrictions? In the context of Australia with soon-to-be-reached 80% vaccination targets, and hence the relative reduction in the lockdown benefits, we are of the opinion that it warrants the transition away from lockdown-centered policies.

## Author Contributions

AP conceived the idea, wrote the draft, and revised the paper. OA wrote the draft and revised the paper. EM conceived the idea and revised the paper. All authors contributed to the article and approved the submitted version.

## Conflict of Interest

The authors declare that the research was conducted in the absence of any commercial or financial relationships that could be construed as a potential conflict of interest.

## Publisher's Note

All claims expressed in this article are solely those of the authors and do not necessarily represent those of their affiliated organizations, or those of the publisher, the editors and the reviewers. Any product that may be evaluated in this article, or claim that may be made by its manufacturer, is not guaranteed or endorsed by the publisher.

## References

[1] Pak A Adegboye OA. The importance of structural factors in COVID-19 response in Western Pacific. Asia Pac J Public Health. (2021) 33:977–8. 10.1177/10105395211035932 34315241

[2] Adekunle A Meehan M Rojas-Alvarez D Trauer J McBryde E. Delaying the COVID-19 epidemic in Australia: evaluating the effectiveness of international travel bans. Aust N Z J Public Health. (2020) 44:257–9. 10.1111/1753-6405.13016 32697418 PMC7405030

[3] Boyd M Baker MG Wilson N. Border closure for island nations? Analysis of pandemic and bioweapon-related threats suggests some scenarios warrant drastic action. Aust N Z J Public Health. (2020) 44:89. 10.1111/1753-6405.12991 32259383 PMC7262147

[4] Cornwall W. Can you put a price on COVID-19 options? Experts weigh lives versus economics. Science. (2020). 10.1126/science.abb9969

[5] Thunström L Newbold SC Finnoff D Ashworth M Shogren JF. The benefits and costs of using social distancing to flatten the curve for COVID-19. J Benefit Cost Analy. (2020) 11:179–95. 10.1017/bca.2020.1230886898

[6] Gupta A Zhu H Doan MK Michuda A Majumder B. economic impacts of the COVID−19 lockdown in a remittance-dependent region. Am J Agric Econ. (2021) 103:466–85. 10.1111/ajae.12178

[7] Umer H Khan MS. Evaluating the effectiveness of regional lockdown policies in the containment of COVID-19: evidence from Pakistan. arXiv. (2020).

[8] Miles DK Stedman M Heald AH. Stay at Home, Protect the National Health Service, Save Lives: a cost benefit analysis of the lockdown in the United Kingdom. Int J Clin Pract. (2021) 75:e13674. 10.1111/ijcp.13674 32790942 PMC7435525

[9] Mulligan CB Murphy KM Topel RH. Some Basic Economics of COVID-19 Policy: A Look at the Trade-Offs We Face in Regulating Behavior During the Pandemic. Chicago Booth Review (2002). Available online at: https://review.chicagobooth.edu/economics/2020/article/some-basic-economics-covid-19-policy (assessed September 22, 2021).

[10] Allen DW. COVID Lockdown Cost/Benefits: A Critical Assessment of the Literature. (2021). Available online at: http://www.sfu.ca/~allen/LockdownReport.pdf (assessed September 22, 2021).

[11] Acemoglu D Chernozhukov V Werning I Whinston MD. Optimal Targeted Lockdowns in a Multi-Group SIR Model. National Bureau of Economic Research Working Paper No. w27102 (2020). Available online at: https://www.nber.org/system/files/working_papers/w27102/w27102.pdf (assessed September 22, 2021).

[12] Gros C Valenti R Schneider L Valenti K Gros D. Containment efficiency and control strategies for the Corona pandemic costs. Sci Rep. (2021) 11:1–13. 10.1038/s41598-021-86072-x 33767222 PMC7994626

[13] Scherbina A. Assessing the optimality of a COVID lockdown in the United States. Econ Disasters Clim Change. (2021) 5:177–201. 10.1007/s41885-021-00083-6 33997600 PMC8105692

[14] Singh AK Gillies CL Singh R Singh A Chudasama Y Coles B. Prevalence of co-morbidities and their association with mortality in patients with COVID-19: a systematic review and meta-analysis. Diabetes Obes Metab. (2020) 22:1915–24. 10.1111/dom.14124 32573903 PMC7361304

[15] Levere M Rowan P Wysocki A. The adverse effects of the COVID-19 pandemic on nursing home resident well-being. J Am Med Directors Assoc. (2021) 22:948–54.e2. 10.1016/j.jamda.2021.03.010 33861980 PMC7980137

[16] Cutler D M Summers LH. The COVID-19 pandemic and the $16 trillion virus. JAMA. (2020) 324:1495–6. 10.1001/jama.2020.19759 33044484 PMC7604733

[17] Robinson LA Sullivan R Shogren JF. Do the benefits of COVID-19 policies exceed the costs? Exploring uncertainties in the Age–VSL relationship. Risk Anal. (2021) 41:761–70. 10.1111/risa.13561 32677076 PMC7405126

[18] Brown N Te Riele K Shelley B Woodroffe J. Learning at Home During COVID-19: Effects on Vulnerable Young Australians. Independent Rapid Response Report. Hobart: University of Tasmania, Peter Underwood Centre for Educational Attainment (2020). Available online at: http://www.wscf.org.au/wp-content/uploads/2020/06/Learning-at-home-during-COVID-19-Effects-on-vulnerable-young-Australians.pdf (assessed September 22, 2021).

[19] Headspace. Insights: Youth Mental Health and Well-Being Over Time. (2020). Available online at: https://headspace.org.au/assets/Uploads/Insights-youth-mental-health-and-wellbeing-over-time-headspace-National-Youth-Mental-Health-Survey-2020.pdf (assessed September 22, 2021).

[20] Browning MH Larson LR Sharaievska I Rigolon A McAnirlin O Mullenbach L. Psychological impacts from COVID-19 among university students: risk factors across seven states in the United States. PLoS ONE. (2021) 16:e0245327. 10.1371/journal.pone.0245327 33411812 PMC7790395

[21] Pak A McBryde E Adegboye OA. Does high public trust amplify compliance with stringent COVID-19 government health guidelines? A multi-country analysis using data from 102,627 individuals. Risk Manag Healthc Policy. (2021) 14:293. 10.2147/RMHP.S278774 33542664 PMC7851580

[22] Lally M. The Costs Benefits of COVID-19 Lockdowns in Australia. (2021). Available online at: https://clubtroppo.com.au/files/2021/04/THE-COSTS-AND-BENEFITS-OF-A-COVID-LOCKDOWN-6.pdf (assessed September 22, 2021).

[23] Goldstein JR Lee RD. Demographic perspectives on the mortality of COVID-19 and other epidemics. Proc Natl Acad Sci. USA. (2020) 117:22035–41. 10.1073/pnas.2006392117 32820077 PMC7486771

[24] Dawel A Shou Y Smithson M Cherbuin N Banfield M Calear AL. The effect of COVID-19 on mental health and well-being in a representative sample of Australian adults. Front Psychiatry. (2020) 11:619331. 10.3389/fpsyt.2020.619331 33551879 PMC7860974

